# Modern Wound Dressings: Hydrogel Dressings

**DOI:** 10.3390/biomedicines9091235

**Published:** 2021-09-16

**Authors:** Valentin Brumberg, Tatiana Astrelina, Tatiana Malivanova, Alexander Samoilov

**Affiliations:** Burnasyan Federal Medical Biophysical Center of the Federal Medical Biological Agency, 123098 Moscow, Russia; brumb1225@gmail.com (V.B.); tmalivanova@yandex.ru (T.M.); asamoilov@fmbcfmba.ru (A.S.)

**Keywords:** acute wounds, chronic wounds, semipermeable film dressings, semipermeable foam dressings, hydrocolloids, hydrogels

## Abstract

Chronic wounds do not progress through the wound healing process in a timely manner and are considered a burden for healthcare system; they are also the most common reason for decrease in patient quality of life. Traditional wound dressings e.g., bandages and gauzes, although highly absorbent and effective for dry to mild, exudating wounds, require regular application, which therefore can cause pain upon dressing change. In addition, they have poor adhesional properties and cannot provide enough drainage for the wound. In this regard, the normalization of the healing process in chronic wounds is an extremely urgent task of public health and requires the creation and implementation of affordable dressings for patients with chronic wounds. Modern wound dressings (WDs) are aimed to solve these issues. At the same time, hydrogels, unlike other types of modern WDs (foam, films, hydrocolloids), have positive degradation properties that makes them the perfect choice in applications where a targeted delivery of bioactive substances to the wound is required. This mini review is focused on different types of traditional and modern WDs with an emphasis on hydrogels. Advantages and disadvantages of traditional and modern WDs as well as their applicability to different chronic wounds are elucidated. Furthermore, an effectiveness comparison between hydrogel WDs and the some of the frequently used biotechnologies in the field of regenerative medicine (adipose-derived mesenchymal stem cells (ADMSCs), mesenchymal stem cells, conditioned media, platelet-rich plasma (PRP)) is provided.

## 1. Introduction

Impairment of a normal wound healing process and consequently chronic hard-to-heal wound formation are the most common reasons for the decrease in patients’ quality of life also for their disability., An estimated abundance of chronic wounds with mixed etiology is 1–2% in general population, that is compatible with abundance or cardiovascular system diseases [1,2]. 

Acute wounds could be repaired by a normal wound healing process for two or three months, depending on the size and depth of the damaged skin tissue [3]. A chronic wound propagation leads to significantly more serious ramifications because of the impairments on normal wound healing caused by a complex of pathogenetic factors. Chronic wound injuries include infected wounds, leg and pressure ulcers, burns of various origins, and diabetic foot syndrome [3]. The care of such wounds is an extremely urgent task of public healthcare and requires the creation and implementation of coatings available for patients with these chronic wounds.

**Figure 1:**
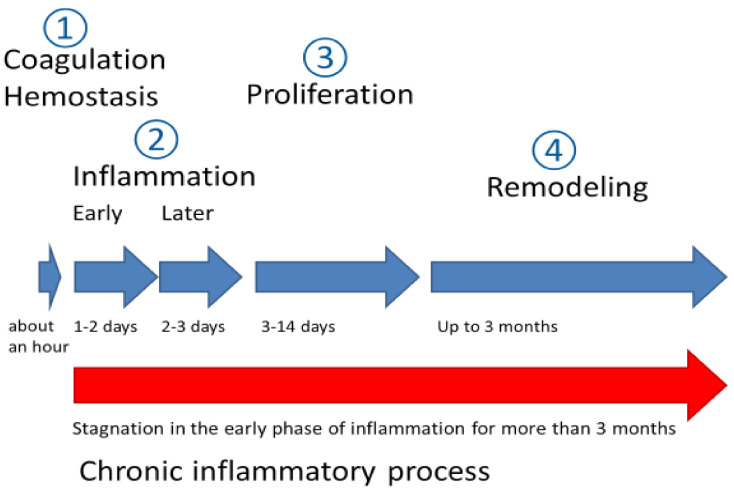
Acute wound healing stages (blue) and stagnation of the inflammatory process that results in chronic wound formation (red).

Wound healing is a dynamic and complex process of tissue regeneration and growth that includes four stages (Figure 1) [4]. The first one is the coagulation and hemostasis that begins from the microvascular bed injury and includes fibrin clot formation, degranulation and aggregation of [5] thrombocytes (PDGF, VEGF and many others). As a consequence, this leads to involvement of fibroblasts, macrophages and endothelial cells in wound repair [5]. The second, inflammatory stage, is accompanied by infiltration of the wound and its surrounding tissue with inflammatory cells—granulocytes (specifically, fragmented nuclei neutrophils) during the 24–48 h after injury and monocytes 48–72 h after injury. Next, lymphocytes migrate to the wound area recruited by IgG and interleukin 1 IL-1). The third stage, proliferation, continues from three days to two weeks after injury, followed by fibrin scaffold replacement by a newly formed granulation tissue as a result of extracellular matrix components (collagens types I and III, laminin 1, nidogen) and glycosaminoglycans synthesis by fibroblasts as well as auto- and paracrine actions of fibroblasts. The final stage is a remodeling which is governed by an equilibrium by tissue matrix metalloproteinase (MMP) proteolytic action and their corresponding tissue inhibitors (TIMPs). During this remodeling stage, the diameter of the collagen fibers increases in addition to a decrease in hyaluronic acid and other glycosaminoglycans content relative to collagen [4,5,6]. As a result of the normal wound healing process a dense scar tissue is formed, characterized by an absence of cells and vessels. 

Unlike acute wounds, the healing of chronic wounds or ulcers usually stagnates in the early inflammation stage for three months after the injury without reaching proliferation and subsequent healing (Figure 1). Key features of chronic wounds include: a prolonged inflammatory stage; excessive infiltration of neutrophils into the wound area; the presence of persistent infections [7]; the predominance of the matrix metalloproteinase (MMP) levels over the corresponding tissue inhibitors [8]; and fibroblasts in chronic wounds and ulcers differ in phenotype and have a reduced migration capacity compared to acute wounds.

In particular, dramatically reduced levels of the expression of transforming growth factor receptors (TGFβ) in biopsies from non-healing edges of venous ulcers have been reported [8]. It should be noted that routinely used traditional wound gauze dressings are intended mainly for dry wounds (for example, to facilitate the autolytic process of scab removal) and, despite their ability to absorb exudate and drain the wound, require constant replacement to prevent maceration and adhesion to the wound surface [9]. The latter circumstance potentially contributes to the painfulness of changing the dressing. In this regard, the normalization of the healing process in chronic wounds is an extremely urgent task of public health and requires the creation and implementation of affordable dressings for patients with chronic wounds. In comparison with traditional WDs, modern ones provide the wettability of the wound surface, gas exchange, exudate absorption, are non-adhesive to the wound surface, and are able to enhance autolytic wound debridement. Among modern WDs, hydrogel materials occupy a special niche due to the wide potential as systems for targeted delivery of drugs [10], antibiotics [11], nanoparticles [12], growth factors [13] and regulatory peptides [14]. This mini review briefly discusses both traditional and modern WDs, among which special attention is paid to hydrogels and hydrocolloids, and, in addition, the classification of hydrogels by the mechanism of gelation provided. Bioactive coatings, tissue-engineered skin equivalents, are mentioned cursorily. 

**Figure 2:**
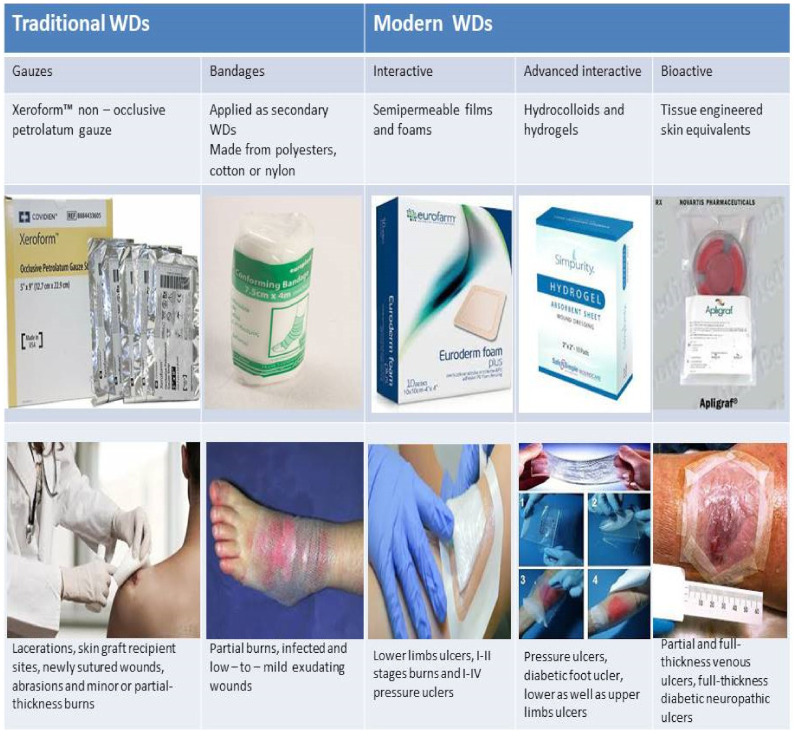
Briefly description of WDs types with examples.

Wound dressing classifications used in this manuscript are presented below in Figure 2; for each type of dressing suitable wounds are briefly named. 

## 2. Types of Wound Dressings (WDs)

Traditional WDs belong to passive dressings and are usually applied to dry and well-cleaned wounds and include gauzes and bandages. Gauze dressings are presented by woven and non-woven cotton fibers, viscose, and polyesters. The main function of these dressings is an exudate and fluid absorption from an open wound due to their fibrous structure. For example, Xeroform ™ (non-occlusive dressing) is a petrolatum gauze impregnated with 3% bismuth tribromo phenate and is used to cover dry or exudative mild wounds [15]. Bandages are applied as secondary WDs made from natural cotton, wool, cellulose, rayon, polyester, or polyamide. It should be highlighted that cotton bandages could be used for wound cleaning but they shed fibers and stick to the wound surface; they are generally used for dry venous and arterial ulcers. Rayon, polyester and polyamide bandages are non-adherent absorbent secondary dressings that are permeable for liquid and water vapour that do not stick to the wound surface and hence are suitable for granulated wounds with a mild to moderate exudate. 

Since traditional WDs cannot provide enough drainage of the wound, they have been replaced by modern dressings, which are characterized by semi-permeability and the presence of a highly absorbent layer. In addition, modern WDs accelerate the formation of granulation tissue and facilitate the migration of epithelial cells from the edges of the wound to its center [16]. 

Since each individual type of chronic wound is characterized by different etiologies and pathologies of healing, it becomes necessary to understand the rationale of using particular dressings, depending on the type of wound and the stage of the wound process (Table 1).

Modern WDs are usually semi-occlusive or occlusive and presented mainly by synthetic polymers and divided into interactive, advanced interactive, and bioactive catergories [17,18]. Interactive dressings include semi-permeable films and foams, advanced interactive dressings are presented by hydrocolloids and hydrogels, while tissue-engineered skin equivalents belong to bioactive WDs [19,20].

Semi-permeable film dressings consist of a porous transparent and adhesive polyurethane which provides an aqueous vapor transmission, O_2_ and CO_2_ gas exchange, autolytic scab removal, and, in addition, polyurethane films that are impermeable to bacteria [10]. Initially, similar films were made from nylon with adhesive polyurethane edges, while polyurethane was used as a backing to impart compression properties [13]. Despite the disadvantages of nylon dressings, which include insufficient absorptive capacity for them to be applied to wounds with profuse exudation, such dressings are highly elastic and can be applied to the surface of any shape; in addition, the transparency of the nylon and polyurethane film makes it possible to monitor wound healing without removing the dressing. Wound dressings of this category are used as secondary dressings to cover primary gelling dressings (Hydrofilm / Hydrofilm ™).

These dressings are used for superficial wounds and abrasions and include FDA - approved Opsite ™, Tegaderm ™, Biooclusive ™, and Hydrofilm / Hydrofilm ™ [21].

Semi-permeable foam dressings are composed of a hydrophobic polyurethane film and a hydrophilic wound-facing foam. This category includes dressings based on polyurethane (Askina Foam ™, Lyofoam ™, Allevyn ™ and Tielle ™) [22].

Such dressings are used in the treatment of ulcers of the lower limbs and are indicated for I-II stage burns and I-IV stage pressure ulcers. It should be noted that these dressings are used only as primary ones due to the good permeability of polyurethane foam to water vapour [23]. However, the main disadvantages of dressings based on polyurethane foam are the need for frequent replacement and unsuitability for dry wounds and dry scars, since the effectiveness of these dressings directly depends on the course and severity of the exudative process.

**Table 1:** Summary of the applicable dressings for mixed etiology chronic wounds (from C.Shi et al., 2020 [24]).

Wound Type	Etiology	Properties	Applicable Dressing (Example)
Diabetic foot ulcer	Neuropathy and lower limb diseases	Insufficient oxygen and blood supply to the wound bed; healing stagnates in the inflammation stage Weak, moderate, or profuse exudation	Semipermeable non-adhesive and adhesive seals presented by foams and hydrocolloids Examples: UrgoStart contact dressing Allevyn, Biatain and Tegaderm dressings
Pressure ulcers	Local ischemia and tissue injury	Local injury of skin or subcutaneous fat Low–to–moderate exudation	Semipermeable non-adhesive foam dressings and hydrocolloids. Examples: Mepilex Ag, DuoDerm, SignalTM, and DuoDerm ExtraThin
Burns	Thermal, chemical or radiation skin injuries	Propensity to secondary infection, wounds with profuse exudation potentially extending to dermal layers, subcutaneous fat, muscles and bone tissue	Occlusive dressings with high absorptive capacities Examples: alginate, chitosane, collagen, hyaluronic acid hydrogels or fibrous dressings able to form a gel under a contact with wound surface (carboxymethylcelluloseHydrofiber, Algisite M, HemCon Bandage Pro, Hydrofiber)
Chronic venous ulcers	Lower limb vascular diseases	Blood supply disturbance; pronounced formation of necrotic tissue, abundant exudation on ulcer surface, accompanied by multiple infections	Semipermeable foam dressings Examples: Mepilex, Allevyn, Contreet Ag, Coloplast
Radiation dermatitis	Local radiation induced skin injury	Impairment of wound healing in proliferation stage and consequent alteration of granulation tissue	Film barrier dressings in form of gauze or spay Examples: 3MTM Cavilon, SECURA, Medi Derma S

Hydrocolloids are interactive occlusive dressings consisting of two layers, the inner one, which is presented by a suspension of hydrophilic colloidal particles, and the outer polyurethane layer, which is impermeable to bacteria. Thus hydrocolloids are a combination of gel-forming agents (carboxymethylcellulose, gelatin and pectin) with other materials such as elastomers and adhesive coatings [16]. The principle of action of hydrocolloids is based on the formation of a gel phase upon contact with the wound surface, which helps to moisturize the wound and protect the granulation tissue due to the absorption of exudate by the dressing material. Dressings such as Granuflex ™, Comfeel ™, and Tegasorb ™ are available as sheets or thin films [25]. Hydrocolloids are prescribed for full-size and partial wounds with low to medium exudation, wounds with scab formation and that can remain on the wound surface for up to seven days [26].

Lee et al. [27] developed a hydrocolloidal covering material by coating a polyurethane film with a mixture of carboxymethyl cellulose and styrene-isoprene- styrene with the addition of silk fibroin nanoparticles. In order to study the effects of the obtained hydrocolloid on the healing of burn wounds, deep second-degree burns of 1.0 - 1.5 cm were created in eight adult Sprague Dawley rats by heating the dorsal region to 60 ° C for 30 s. The animals were divided into three groups depending on the coating used - hydrocolloid, commercial Neoderm dressing, and gauze (control). Observation of the animals was carried out for three weeks after the creation of the burn. According to the observation results, it was shown that the area of the residual wound surface on the 14th and 21st days of observation was significantly lower in the group with the hydrocolloid compared to the commercial coating, Neoderm, and control [27].

The disadvantages of hydrocolloids are limited applicability; these dressings are not prescribed for neurotrophic ulcers and wounds with abundant exudate [28]. In addition, some hydrocolloid coatings have been reported to adhere to the wound bed and, as a consequence, are difficult to remove [29].

HydrogelWDs. The advantage of hydrogels over traditional wound dressings is their ability to adapt the procurement depending on the wound and the stage of the wound process [30]. Hydrogels meet most of the criteria for modern wound dressings [31]: the ability to absorb wound exudate; the maintaining of a moist environment; the maintaining of gas exchange; thermal insulation; the presence of antibacterial properties; safety; ease of removal from the wound surface, painlessness of changing the dressings for the patient; ease of handling for the operating surgeon; biomechanical and viscoelastic properties (storage and loss moduli, suture retention strength) adequate for suturing to the wound surface or applying to the wound; and a simple and reliable sterilization method.

Hydrogels can be defined as highly hydrated polymer materials (>30% water by weight) whose structural integrity is ensured by physical and chemical intermolecular crosslinks between polymer chains [32] or as polymer networks that exhibit the ability to swell and retain a significant volume of water but are water insoluble [33]. Thus, hydrogels as a type of interactive WDs are used for uninfected low exuding wounds; however, depending on the ability of the gel to swell, they can be used to cover moderately or profusely exuding wounds. It is noted that hydrogels are often used to treat burns and ulcers and, in addition, can facilitate autolysis and removal of scabs and can be used to cleanse wounds [34]. However, hydrogel wound dressings in most cases require the application of a secondary dressing to ensure adhesion to the wound bed (e.g., a hydrocolloid). In addition, these dressings require regular replacement at intervals of two to three days; therefore, hydrogel dressings can be non-adhesive (alginate, gelatin, carboxymethyl cellulose) or completely biodegradable.

The classification of hydrogels in the context of their preparation is discussed in detail below.

There are two main approaches to the classification of hydrogels. By their origin, hydrogel polymer networks can be synthetic (polyethylene oxide (PEO), polyvinyl alcohol (PVA), polyacrylic acid (PAA), polypropylene fumarate-co-ethylene glycol (P (PF-coEG), PEO-PEG-PEO, etc.), natural (alginate, chitosan, collagen, gelatin, hyaluronic acid, gellan gum, polyhydroxybutyrate valerate, cellulose, fibrin, etc.), and composite [35].

On the other hand, hydrophilic polymer networks can be formed through both covalent and supramolecular non-covalent interactions, and from this point of view, hydrogels can be classified according to the method of functionalization (introduction of additional functional groups) and the polymerization mechanism (hydrogels with physical, chemical and physicochemical gelation mechanisms). The latter category also includes interpenetrating polymer networks [36]. Hydrogels with a purely physical mechanism of gelation do not require the introduction of additional functional groups for polymerization, but they are characterized by weak viscoelastic properties [36,37], which are difficult to control. Even though hydrogels with physical gelation are pseudoplastic, their use is limited to applications not associated with deformation loads such as, for example, an injection hydrogel [38]. It should be noted that hydrogel dressings routinely used in clinical practice have a physical mechanism of gelation or consist of interpenetrating polymer networks (for example,; Helix3 cm-highly porous flat sheets of type I collagen; Algisite M - calcium alginate dressing) [39]. On Table 2 there is a list of the main physical non – covalent interactions used for hydrogel polymerization. 

On the other hand, hydrogels with a chemical mechanism of gelation are characterized by more satisfactory viscoelastic properties, which determine their use as biomaterials with increased resistance to degradation, as well as dressing materials. In addition, the rheological properties, along with the degradation kinetics, depend on the concentration of hydrogel precursors and the density of the forming bonds; however, the nature of the bonds themselves has a weak effect on the mechanical properties [40].

Biodegradation of hydrogels with chemical polymerization is dependent not only on the nature of the biopolymer but also on stability of crosslinking bonds. For instance, the biodegradable hydrogels were obtained by use of MMP-specific peptide linkers and it was shown that degradation of these hydrogels is matched with cell growth and spreading in culture [43]. Flexible degradation kinetics mediates an application of hydrogels as delivery vehicles for drugs or as biological active substances. A light-responsive hydrogel platform for growth factors release was studied by Zhang et al. [44] who dispersed liposomes with black phosphorous quantum dots in F127 gel containing granulocyte macrophage – colony stimulating factor (GM-CSF). The heat generated by black phosphorus (BP) under 808 nm near-infrared laser irradiation accelerates the F127 gel ablation and the release of GM-CSF respectively.

On Table 3. a list of reactions used for the chemical polymerization of hydrogels is given. Such crosslinking reactions are conventionally called “bioorthogonal”, because all components used for polymerization (precursors, cofactors, initiators, etc.) participate only in the formation of the polymer network, but do not interact with biomolecules [45].

An antibacterial effect of cellulose hydrogels loaded withsilver nanoparticles, was shown for gram—positive and negative bacteria [46]. Nešović et al. introduced an effective method for the development of bandaging tools with improved properties based on the biocompatible chitosan-polyvinyl alcohol hydrogels with silver nanoparticles embedded. They have shown the absence of cytotoxic action as well prominent antibacterial activity against S. aureus и *E. coli* for these hydrogels.

Sun et al. [47] synthesized a photopolymerizable hydrogel including an antibacterial polypeptide ε-polylysine by a functionalization of ε-polylysine (ε-PL) and γ-poly(glutamic acid) with glycidyl-methacrylate (GMA) with a formation of ε-polylysine-glycidyl methacrylate (ε-PL-GMA) and γ-poly(glutamic acid)-glycidyl methacrylate (γ-PGA-GMA). The showed that hydrogels containing ε-PL-GMA demonstrated high levels of activity against *S. aureus* and *E. coli* and reduced the bacterial levels to lower than 10^3^ CFU/mL, contrary to hydrogels without ε-PL-GMA. Z.Abdollahi et al. [48] studied the antibacterial effect of sodium carboxymethylated starch (CMS) hydrogel-containing CuO nanoparticles against Gram-positive and gram-negative bacteria. *Listeria monocytogenes*, *Enterococcus faecalis*, *Staphylococcus aureus*, methicillin-resistant *Staphylococcus aureus*, *Salmonella enterica*, *Pseudomonas aeruginosa*, *Escherichia coli*, and *Yersinia enterocolitica* microbial suspensions were spread on Muller Hinton Agar. After CuO NPs, CMS, CMS-2%CuO, and CMS-4%CuO were inoculated on the MHA surface to enable the diffusion of agents. Bacterial growth inhibition was assessed by measuring the diameter of inhibition zones. It was found that copper oxide nanoparticles also exhibited antibacterial activities against the eight bacterial species, with the diameter of inhibition zones between 20 mm and 32 mm.

Kong et al. [49] obtained the alginate hydrogel-containing bioglass and deferoxamine and this hydrogel effectiveness was confirmed for the streptozocin-induced diabetes wound model. Dong et al. [50] developed an improved method for the delivery of adipose tissue-derived mesenchymal for the treatment of burn wounds, which consists of the use of an in situ polymerizing system formed by a branched polyethylene glycol diacrylate, thiol-hyaluronic acid, and a short RGD peptide. In another study published by S. Grijalvo et al. [51], epidermal growth factor-loaded liposomes were introduced into the chitosan hydrogel, and an effect of the resulting hydrogel system was evaluated for second-stage burn wounds in rats. An immunohistochemistry study showed a significant increase in cell proliferation and epithelialization rate.

Hydrogel WDs are indicated mainly for burn wounds from moderate to profuse exudation due to their high absorbency and hydrophilicity. For example, an application of a hydrogel dressing for thermal burn coverage in the Sprague Dawley rats model has shown a significant improvement in wound closure, re-epithelialization, improved cosmetic appearance, and a decrease in fibrosis in comparison with control animals [52]. Another study [53] showed a positive effect of a composite hydrogel of collagen and hyaluronic acid on human microvascular endothelial cells’ (HMEC) and COS7 fibroblasts’ proliferative activity. Additionally, in the same study a decrease in the full-size wounds area (with an initial diameter of 8 mm) was shown for adult ICR mice when using collagen hydrogel and hyaluronic acid as an adhesive dressing. In addition, on the 14 h day after an full thickness wound introduction by excision in the back and abdomen, the granulation tissue with a thickness up to 1300 µm was observed [53]. Wang et al. obtained a hydrogel composed of modified hyaluronic acid, dextran and β-cyclodextrin with the inclusion of the drug resveratrol and a VEGF encoding plasmid that mediated a pro-angiogenic and anti-inflammatory effect for burn wounds in rats [54].

Tissue-engineered skin equivalents refer to bioactiveWDs. These include cultured skin substitutes mainly consisting of layers of keratinocytes and fibroblasts with an underlying layer of collagen (ApliGraf^®^), such as polyglactin, nylon mesh (Dermagraft^®^, TransCyte^®^), composites of an acellular dermal matrix, and components of human or animal origin (AlloDerm^®^, AlloMax^®^, GraftJacket^®^, Integra^®^ etc.). Cellular products are prescribed for non-infected partial and full-thickness venous ulcers, full-thickness diabetic neuropathic ulcers, as well as diabetic foot ulcers [55]. Acellular dermal scaffolds are used in reconstruction of complex surgical defects. Although bioactive WDs are FDA-approved and biocompatible, acellular dermal scaffolds cannot mediate sufficient vascularization, and there are some safety concerns associated with infection transmission in allotransplantation. With regard to cultured skin substitutes, their procurement is expensive and time-consuming, after cell isolation and expansion, the time required for culture and maturation of cultured cell substitutes is 45 days [56].

## 3. Discussion

New technologies in chronic wound treatment, from which adipose-derived mesenchymal stem cells (ADMSCs), mesenchymal stem cells-conditioned medium, platetet-rich plasma (PRP) in combination with hyaluronic acid scaffold or fat grafts - can be used conjointly with modern WDs to address different stages of wound healing. Among all aforementioned WDs, hydrogels are of particular importance since they play a pivotal role in the delivery of bioactive molecules and cellular products to the injury site, unlike other types of modern WDs. In this section a comparison between the effectiveness of new biotechnologies in wound healing and hydrogel WDs is presented.

ADMSCs are particularly important for wound healing since they can be relatively easily obtained from liposuction waste, have the advantage of immunocompatibility, and are more suitable for allotransplantation. ADMSCs can differentiate into keratinocytes, fibroblasts and endothelial cells [57]. Regarding their paracrine action, they secrete a vast profile of cytokines and growth factors including fibroblast growth factor, VEGF, hepatocyte growth factor, interleukin (IL)-6, IL-8, granulocyte colony-stimulating factor, platelet-derived growth factor AA (PDGF-AA), and granulocyte–macrophage colony-stimulating factor [58]. Through the action of these secreted proteins, ADMSCs can modulate the activities of cells recruited in the proliferation phase of wound healing, e.g., endothelial cells and fibroblasts. For example, according to Zhao J. et al. (2013) [59], VEGF, bFGF, and PDGF-AA can promote the migration of fibroblasts, while bFGF and EGF can promote the proliferation of fibroblasts. ADMSCs as well as their products can effectively promote wound repair: for instance, ADMSCs; secreted exosomes can promote the proliferation and migration ability of fibroblasts in a dose-dependent manner as well as significantly stimulate the expression of collagens types I and III by fibroblasts in vitro [60]. However, there are some concerns in the literature on the effectiveness of ADMSCs as theurapetic agents alone or in combination with a hydrogel scaffold. For instance, H.Ma (2021) [61] locally injected excisional wounds in diabetic rats with ADMSCs and found wound healing rates are almost comparable to those of normal rats. In another study, Ting-Yu Lu et al. [62] compared the effectiveness of gelatin/microbial transglutaminase (mTG) hydrogel with encapsulated ADMSCs spheroids in a murine thermal burn wound model with other treatment groups: mice with the hydrogel only, mice with the cell-suspension encapsulated in hydrogel, and a group with ADMSCs injected without a hydrogel. It was shown that the group treated with ADMSCs-spheroids embedded into the hydrogel achieved the highest wound contraction rate of 55.3%, followed by cell suspension with hydrogel (45,2%), hydrogel only (37.1%), cell suspension (32.3%), and control (30.2%) on day 14 [62]. Furthermore, these authors have shown a reduced discoloration rating at day 10 for groups with ADMSCs-spheroids encapsulated hydrogel and hydrogel with cell suspension. Besides the discoloration rating reduction was statistically significant between those two groups (ADMSCs – spheroids in hydrogels, ADMSCs suspension in hydrogel) and groups received ADMSCs or hydrogel separately [62].In another study conducted by M.Yang et al. (2020) [62] the effectiveness of hydrogels chitosan/collagen/β-glycerolphospate, alone and in combination with placental MSCs spheroids and placental MSCs in suspension, was compared in db/db mice. Animals were divided into three groups: chitosan/collagen/β-glycerolphospate hydrogel alone (control), group received chitosan/collagen/β-glycerolphospate hydrogel with MSCs spheroids and chitosan/collagen/β-glycerolphospate with placental MSCs suspension. Observation of wounds at weeks 3, 10 and 14 in ADMSCs spheroid-encapsulated chitosan/collagen/β-GP hydrogel-treated db/db mice found almost complete wound closure compared with the control group and the ADMSCs suspension encapsulated chitosan/collagen/β-GP hydrogel-treated group [62].

*Human mesenchymal stem cells-conditioned medium (MSCs-CM)* contains cytokines and growth factors that can facilitate the regeneration and repair of various tissues and organs. For example, Robert A.W. (2019) [63] studied the effectiveness of a conditioned medium prepared from skin-derived mesenchymal stem cells (SD-MSCs) in combination with carrageenan (CG) and polyvinylalcohol (PVA) hydrogels for cutaneous wound healing in C57BL/6 mice in which a full thickness excision with cuts 6 mm in diameter was created. The mice were subdivided into six experimental groups: control, CG hydrogel, CG hydrogel-embedded with SD-MSC-conditioned medium, PVA hydrogel, PVA hydrogel-embedded with SD-MSC-conditioned medium, and SD-MSC-conditioned medium. Wounds were photographed on days 3 and 14 days and their diameters were counted using ImageJ software. It was shown that on the third day a significant wound size decrease was noticeable in animals treated with hydrogels in comparison with control group (mice without any treatment). On day 14 post treatment all groups demonstrated a decreased wound surface area; however in group treated with PVA hydrogel with a conditioned medium from SD-MSCs the larger wound areas where observed which is associated with the lack biocompatibility of PVA [64]. Platelet-rich plasma (PRP) and its different modes of application. Platelet-rich plasma (PRP) is an autologous blood-derived product enriched in platelets, growth factors, chemokines and cytokines. It is a reservoir of essential growth factors including platelet-derived growth factor, vascular endothelial growth factor, transforming growth factor-beta 1, and insulin-like growth factor, which facilitates repair and healing. PRP can be autologously activated or autologous non-activated [65]. PRP can be used for chronic wound healing in combination with hydrogels or on their own; for example, Semenič D, (2018) [66] conducted a clinical prospective study on safety and efficacy of the platelet gel (form of PRP activation) and hydrogel (3 M Tegaderm, USA). Sixty patients (42 males and 18 females, mean age 69.43 years, SD 14.74) with chronic lower leg ulcers of different etiologies were treated, half with allogenic platelet gel and half with hydrogel. The authors have shown that healing of chronic wounds with the platelet gel was more effective compared to the treatment with hydrogel.

In another study [67], 364 patients with chronic venous leg ulcers were divided into two groups, one that received PRP in combination with hyaluronic acid and the other treated with hyaluronic acid (HA) alone. After 30 days the PRP + HA group showed a statistically significant difference in the re-epithelialization rate in comparison with HA-only treatment, and this difference was still observed even 80 days after treatment.

In a research study conducted by Notodihardjo PV et al. (2014) [68], the efficacy of biodegradable gelatin hydrogel impregnated with PRP was compared with PRP application alone in the murine model of chronic wounds. A total of 180 mice were randomly assigned into one of four groups:the control group, a biodegradable gelatin hydrogel group, a PRP group, and a gelatin hydrogel impregnated with PRP (PRPG) group. They showed that the wound surface area was smaller in the gelatin hydrogel impregnated with PRP group in comparison with the others on days 5, 7 and 14; furthermore, the re-epithelization rate in this group was significantly higher than the others on days 7 and 14. Immunohistochemical staining for von Willebrand factor to confirm neovascularization was conducted on day 14 and it was shown that capillary formation was superior in gelatin-hydrogel impregnated with PRP in comparison with the other two groups of treatment.

## 4. Conclusions

Developing a dressing that considers the factors complicating the normal healing process will greatly benefit patients and wound care professionals. Hydrogels are a promising type of dressing materials due to their porous structure, biodegradability, ability for growth factor incorporation, and controlled release. Among other modern WDs, hydrogels can efficiently and easily be combined with mesenchymal stem cells and as well as their products.. It is noteworthy that results suggest that therapeutic mesenchymal cells (ADMSCs and placenta – derived MSCs) as well as their products perform more efficiently in conjunction with hydrogels: the wound contraction rate is higher in cases of combined application than it is for mesenchymal stem cells or hydrogels alone. As for PRP, there is no strong evidence suggesting a marked advantages of commercially available hydrogel WDs over RPR alone or over its activated form (PRP gel); however, a combined application of PRP with hydrogel WDs can lead to higher rates of wound closure as well as wound re-epithelialization in comparison with PRP or separate hydrogel administration. Taken together, these data suggest the importance of hydrogel WDs application in conjunction with therapeutic agents for efficient wound healing.

## Figures and Tables

**Table 2:** The main physical interaction used for hydrogel polymerization (from C.Echalier et al., 2019 [40]).

Type of Physical Interaction	Exact Binding Mechanism	Example
Guest–host interaction	Interaction between 6,7,8-membered d-glucose (cyclodextrin) units, forming a cavity, with a guest molecule. This interaction is similar to hydrophobic and depends on the geometry of molecules	Cyclodextrin–adamantane
Dynamic protein–protein interactions	Complex interactions, the nature of which is determined by the affinity of the peptide to the protein, the number of repeating units, etc.	WW domain with proline enriched peptide [41]
Hydrophobic interactions “self-assambley”	Manifoldly repeating sequences that provide spiral-spiral interactions (so-called “self-assembly”). Self-assembly is based on a network entropy increase during aggregation of hydrophobic residues inward and exposure of hydrophilic residues in aqueous medium.	Collagen type I XaaYaaGly Gelatin Extracellular tissue matrix hydrogels [42]
Electrostatic interactions		Alginate-Ca^2+^ Heparin-heparin-binding domains of growth factors VEGF, bFGF [42].

**Table 3:** Main chemical reactions used for hydrogel polymerization and cell embedding (from C. Echalier et al., 2019 [40]).

Reaction Type		Reagents Used for Hydrogel Precursors Functionalization	Synthetic or Natural Polymers Used	Embedded Cells
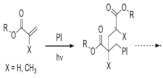	Chain growth radical photopolymerization	Acryloylchloride, methacryolyl chloride, methacrylic anhydride, 2-isocyanatoethyl methacrylate, glycidyl methacrylate	PEG, PLA-PEG-PLA, PVA, chondroitin sulfate, alginate, hyaluronic acid, collagen, chitosan, gelatin	Aorta smooth muscle cells, calvaria osteoblasts, articular chondrocytes, valvuar interstitial cells
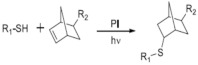	Thiol–ene photopolymerization	5-norbornene-2-carbonic acid, cysteine derivatives, dithiothreitol, 3-mercaptopropionic acid	PEG, gelatin	Mesenchymal stem cells, motor neurons
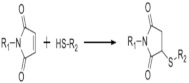	Michael’s addition	N-(2-aminoethyl) maleimide trifluoroacetate, 4-mercaptophenylpropionic acid, mercaptoisobutyrate, 2-dimethyl-3- (4-mercaptophenyl)-propionic acid	PEG, heparin	Pancreatic islets, myoblasts
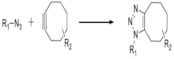	Azide-alkyne cyclocondensation	2-(2-cyclooctin-1-yloxy) acetic acid, bicyclo [6.1.0] non-4-yn-9-ylmethanol or methyl N-succinimidylcarbonate, 11,12-didehydro-5,6-dihydrodibenzo [a, e] cyclooctene-5-ol, 11,12-didehydro-γ-oxodibenz [b, f] azo-cine-5 (6H) -butanoic acid, sodium azide, 4-azidobutanoic acid	PEG	3T3 fibroblasts, mesenchymal stem cells, bone marrow stem cells
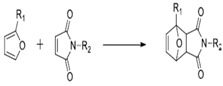	Diels Alder reaction	Furfuryl methacrylate, furfurylamine, 3-(2-Furyl) propanoic acid, N- (2-aminoethyl) male-imide trifluoroacetate salt, 4-(4-N-maleimidophenyl) butyric acid hydrazide, N-maleoyl-β-alanine, N-methoxycarbonylmale-imide	PEG, dextran hyaluronate, poly (N, N-dimethylacrylamide-co-furfuryl methacrylate)	Chondrogenic cells
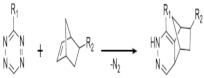	The inverse electron demand Diels Alder reaction	5-[4-(1,2,4,5-tetrazin-3-yl) benzylamino]-5-oxopentanoic acid, 5-norbornene 2-carboxylic acid	PEG, hyaluronate	Mesenchymal stem cell, prostate cancer cells
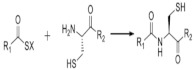	Chemical ligation	5-ethyl-3-mercaptopropionate, Boc-Cys (Trt)-OH	PEG	Insulinoma cells, induced pluripotent stem cells
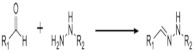	(Acyl)hydrazine formation	Oxalyl chloride / DMSO (Swern oxidation), sodium periodate, tri-Boc hydrazinoacetic acid, hydrazine monohydrate, adipic acid dihydrazide, carbohydrazide, oxalyl dihydrazide	PEG, poly(vinyl alcohol), hyaluronic acid, dexran, carboxymethylcellulose, poly(isopropylacrylamide), poly(aspartic acid), heparin, poly (l-glutamic acid), alginate	Adipose fibroblasts, myoblasts, neuroblastoma cells, vocal cord fibroblasts, neonatal cardiomyocytes
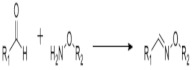	Oxime formation	Sodium periodate, N-hydroxyphthalimide	PEG, hyaluronic acid, alginate	Mesenchymal stem cells, adipose tissue fibroblasts
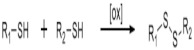	Disulfide bond formation	Dithiobis (propanoic or butyric) dihydrazide, N, N’-bis (acryloyl) cystamine (requires a reduction step), thioacetic acid (requires saponification), N-acetyl-l-cysteine or l-cysteine	Hyaluronic acid, chitosan, PEG, gellan gum, copoly(acrylamide)	-
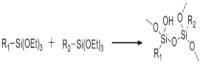	Sol-gel transition	3-isocyanatopropyltriethoxysilane, 3-(glycidoxypropyl) triethoxysilane, 3-aminopropyltriethoxysilane	PEG, gelatin, chitosan, collagen, hydroxypropylmethyl cellulose, alginate	Articular chondrocytes, cardiomyocytes, chondrosarcoma cells, mesenchymal stem cells
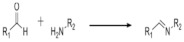	Imine formation	Sodium periodate (oxidative degradation of vicinal diols), 4-formylbenzoic acid, ethylenediamine	Alginate, gelatin, dextran, PEG, chitosan, starch, polyvinylamine, polyphosphasen	Hepatocytes, breast adenocarcinoma, articular chondrocytes, dermal fibroblasts

## Data Availability

Study did not report any data.
